# Intestinal Responses to Short-Term Hexaflumuron Exposure in Grass Carp (*Ctenopharyngodon idella*): A Combined Physiological, Transcriptomic, and Gut Microbiota Perspective

**DOI:** 10.3390/ani16193056

**Published:** 2026-09-28

**Authors:** Shun Zhou, Jiangtao Li, Qiuhong Yang, Xiaohui Ai

**Affiliations:** Yangtze River Fisheries Research Institute, Chinese Academy of Fishery Sciences, Wuhan 430223, China

**Keywords:** hexaflumuron, grass carp, intestinal responses, oxidative stress, transcriptomics, gut microbiota

## Abstract

Hexaflumuron is a benzoylurea insecticide with potential application against arthropod ectoparasites in aquaculture, but its toxicity to non-target fish remains insufficiently characterized. This study evaluated acute toxicity and intestinal responses to sublethal concentrations of hexaflumuron in juvenile grass carp (*Ctenopharyngodon idella*). The 48- and 96-h LC_50_ values were 8.80 and 7.16 mg/L, respectively. Fish were subsequently exposed to nominal concentrations of 0.07 or 0.72 mg/L hexaflumuron for 96 h. Intestinal histopathology, antioxidant biomarkers, 16S rRNA sequencing, and transcriptomics were investigated. Both exposure groups showed focal intestinal alterations. Malondialdehyde levels increased, total superoxide dismutase activity exhibited a biphasic response, and catalase activity and reduced glutathione content declined, indicating altered redox homeostasis. Overall gut microbial structure showed no significant difference, whereas taxon-specific shifts occurred, including an increase in *Aeromonas* to 15.40% at 0.72 mg/L. Transcriptomic analysis identified 1226 and 5496 differentially expressed genes at 0.07 and 0.72 mg/L, respectively. Both concentrations suppressed ribosome biogenesis, protein homeostasis, mitochondrial bioenergetic programs, and complement-associated functions, while the higher concentration induced broader enrichment of cytokine, phagolysosomal, antigen-presentation, and leukocyte-migration pathways. Overall, these results indicated that hexaflumuron perturbs intestinal homeostasis through synergistic impacts on cellular biosynthesis, mitochondrial bioenergetic programs, redox balance, immune regulation, tissue remodeling, and microbial composition.

## Simple Summary

Hexaflumuron is an insecticide with potential use against ectoparasites in aquaculture, but its safety for non-target fish remains uncertain. This study evaluated short-term responses to hexaflumuron exposure in grass carp, an economically important freshwater species. Fish were exposed to 0.07 or 0.72 mg/L hexaflumuron (1/100 and 1/10 of the 96 h LC_50_) for 96 h. Both treatments caused focal intestinal alterations and changes in redox status. Although the overall structure of the intestinal bacterial community remained relatively stable, the abundance of several bacterial groups changed. In particular, potentially opportunistic *Aeromonas* bacteria increased markedly at the higher concentration. Transcriptomic analysis further showed altered protein biosynthesis, cellular energy metabolism, immune responses, and tissue-remodeling pathways. These findings demonstrated that short-term hexaflumuron exposure is associated with intestinal responses at concentrations well below the acute LC_50_. The results provided data relevant to environmental risk assessment and to the further evaluation of treatment conditions in aquaculture.

## 1. Introduction

Benzoylurea (BU) insecticides are a class of insect growth regulators that interfere with chitin biosynthesis and deposition, thereby disrupting molting and metamorphosis in arthropods [1]. Among them, hexaflumuron has been extensively applied worldwide for agricultural pest control because of its high efficacy and selectivity [2]. More recently, its use has expanded into aquaculture as a promising antiparasitic agent [3]. Arthropod ectoparasites, particularly sea lice (*Lepeophtheirus salmonis*) and anchor worms (*Lernaea* spp.), are among the most devastating pathogens in farmed fish, causing severe epidermal injury, secondary bacterial infections, and substantial economic losses [4]. Owing to its arthropod-specific mode of action, hexaflumuron has demonstrated excellent efficacy against sea lice and has been approved for commercial use in major *Atlantic salmon*-producing countries such as Chile, where it is administered as a bath treatment at 2 mg/L (2 ppm) for 1–2 h [5]. In freshwater aquaculture, both patented formulations and our preliminary studies have confirmed its potent antiparasitic activity against *Lernaea* infecting grass carp (*Ctenopharyngodon idella*, Cypriniformes, Xenocyprididae) [6]. Given the widespread occurrence of anchor worm disease in freshwater aquaculture, hexaflumuron represents a promising candidate for ectoparasite control. Moreover, as one of the most economically important freshwater fish species and a natural host of *Lernaea*, grass carp provides an appropriate model for evaluating the safety of the emerging drug in aquaculture [7].

Despite its promise as an antiparasitic treatment, hexaflumuron raises critical regulatory and ecotoxicological concerns. Regulatory authorities have established a maximum residue limit (MRL) of 500 μg/kg for hexaflumuron in fish tissues [3]. In aquatic environments, hexaflumuron exhibits an aqueous hydrolysis half-life of approximately 4–5 days, indicating that residues from therapeutic bath discharges can persist in pond water [8]. Consequently, non-target fish are routinely exposed to residual levels of concentrations following routine treatment, highlighting the need to characterize sublethal effects at environmentally relevant concentrations. However, the toxicological impacts of hexaflumuron on non-target fish species remain poorly understood. Available evidence is limited to a small number of studies focusing primarily on acute toxicity. For example, Ibrahim et al. (2024) have reported a 96 h LC_50_ of 7.19 mg/L in Nile tilapia (*Oreochromis niloticus*), accompanied by behavioral abnormalities, hematological alterations, oxidative stress, immune dysfunction, and tissue damage [9]. Likewise, Soares et al. (2016) found that lufenuron, another representative BU insecticide, induced pathological lesions in the gills and retina of tambaqui (*Colossoma macropomum*) [10]. Although these findings indicated that BU insecticides induce systemic toxicity in fish, available toxicological studies remain largely descriptive and are restricted to tissue-level histopathology and hematological profiles. Consequently, the molecular mechanisms underlying BU-induced toxicity, particularly in specific target organs, remain largely unresolved.

Among these organs, the intestine is particularly vulnerable because it is continuously exposed to waterborne contaminants through drinking and feeding [11]. Beyond its essential roles in nutrient digestion and absorption, the fish intestine serves as a critical mucosal barrier that integrates epithelial integrity, immune defense, and microbial homeostasis. Environmental contaminants can disturb these functions by altering epithelial integrity, redox balance, immune signaling, and gut microbial composition [12,13]. Despite these well-established mechanisms, whether hexaflumuron exerts similar intestinal responses and the molecular pathways involved remain unknown. Combining histopathology, biochemical indices, microbiome, and transcriptomics can connect tissue-level alterations with cellular and molecular responses and thereby provide a more complete assessment of intestinal responses.

In the present study, healthy grass carp were used to evaluate the intestinal responses to short-term hexaflumuron exposure. First, acute toxicity was evaluated using a static exposure assay, and 48 and 96 h LC_50_ values were estimated. Fish were then subjected to a 96 h semi-static exposure at sublethal concentrations corresponding to 1/100 and 1/10 of the 96 h LC_50_. Histopathological examination, antioxidant and lipid-peroxidation assays, 16S rRNA gene sequencing, and intestinal transcriptomics were investigated to characterize the intestinal responses of hexaflumuron in grass carp. These findings provided mechanistic insights into hexaflumuron-induced intestinal responses and offer a scientific basis for the ecological risk assessment.

## 2. Materials and Methods

### 2.1. Experimental Animals

Healthy grass carp (*C. idella*) with a mean body weight of 13.27 ± 1.62 g (sexually immature with undifferentiated gonads) were obtained from a local hatchery and acclimatized under laboratory conditions for 14 days. Fish were maintained in aerated plastic tanks containing dechlorinated water at 24.2 ± 0.5 °C, with a pH of 7.9–8.4, dissolved oxygen of 6.4 ± 0.8 mg/L, and a 12 h light/12 h dark photoperiod. During the acclimation period, fish were fed a commercial pelleted diet (Tongwei Co., Ltd., Tianjin, China; proximate composition: crude protein ≥ 31.0%, crude lipid ≥ 1.0%, crude fiber ≤ 15.0%, crude ash ≤ 12.0%, and moisture ≤ 11.0%) once per day until apparent satiation.

### 2.2. Chemicals and Reagents

Hexaflumuron (purity ≥ 99%, CAS No. 86479-06-3) was purchased from Aladdin Reagent Co., Ltd. (Shanghai, China). A 10 mg/mL stock solution of hexaflumuron was prepared in dimethyl sulfoxide (DMSO, analytical grade) and Tween-80 (analytical grade) at an 8:2 volume ratio. The stock solution was stored at 4 °C in the dark and diluted with ddH_2_O before use. Commercial assay kits for the determination of superoxide dismutase (T-SOD, Cat. No. G4306), catalase (CAT, Cat. No. G4307), reduced glutathione (GSH, Cat. No. G4305), malondialdehyde (MDA, Cat. No. G4302), and total protein concentration (Cat. No. G2026) were obtained from Wuhan Servicebio Bioengineering Institute (Wuhan, China).

### 2.3. Acute Toxicity

Acute toxicity of hexaflumuron was evaluated using a 96 h static exposure test. Based on preliminary experiments, fish were exposed to five nominal concentrations of hexaflumuron (4, 6, 8, 10, and 12 mg/L), together with a blank control group (dechlorinated water only) and a solvent control containing the same concentration of solvent as that used in the highest treatment group. Each treatment consisted of three replicate tanks, with 10 fish per tank. Fish were not fed throughout the exposure period. Mortality was recorded at 24, 48, 72, and 96 h, and fish were considered dead when they showed no response to gentle mechanical stimulation of the caudal peduncle and cessation of opercular movement.

### 2.4. Sublethal Exposure Experiment

Based on the determined 96 h LC_50_, two sublethal exposure concentrations corresponding to 1/10 and 1/100 of the LC_50_ were selected (0.72 and 0.07 mg/L, respectively). Fish were assigned to three experimental groups: high-concentration (0.72 mg/L), low-concentration (0.07 mg/L), and solvent control containing 0.01% solvent. Exposure was conducted for 96 h using a semi-static renewal system, in which the exposure media were completely renewed every 24 h with freshly prepared solutions to minimize temporal variation in nominal hexaflumuron concentrations. Each treatment included three replicate tanks, each containing 20 fish. Water quality parameters, including temperature, dissolved oxygen, pH, and photoperiod, were maintained under the same conditions as those used during acclimatization. Fish were not fed throughout the exposure period.

### 2.5. Sample Collection

At the end of the exposure, six fish per treatment group were randomly selected, anesthetized in an ice-water bath, and immediately dissected for sample collection. For gut microbiota analysis, intestinal content samples were collected using a sterile glass slide, transferred into sterile cryovials, snap-frozen in liquid nitrogen, and stored at −80 °C until microbial DNA extraction. An intestinal segment (approximately 50 mg) was collected, snap-frozen, and stored at −80 °C for transcriptomic analysis. A separate 0.5 cm segment was rinsed with ice-cold phosphate-buffered saline, immediately fixed in 10% neutral-buffered formalin for 48 h, and processed for hematoxylin and eosin staining. The remaining intestinal tissue was rinsed with ice-cold physiological saline, divided into aliquots, snap-frozen, and stored at −80 °C for biochemical assays.

### 2.6. 16S rRNA Gene Sequencing

Microbial genomic DNA was extracted from intestinal samples using the CretMag^TM^ Power Soil DNA Kit (CretBiotech, Suzhou, China) according to the manufacturer’s instructions. DNA quality and concentration were evaluated by 1% agarose gel electrophoresis and a NanoDrop 2000 spectrophotometer (Thermo Fisher Scientific, Wilmington, DE, USA), respectively. The V3-V4 region of the bacterial 16S rRNA gene was amplified using the universal primers 341F (5′-CCTAYGGGRBGCASCAG-3′) and 806R (5′-GGACTACHVGGGTWTCTAAT-3′). Triplicate PCR products were pooled, purified with the AxyPrep DNA Gel Extraction Kit (Axygen Biosciences, Union City, CA, USA), quantified using a Quantus™ Fluorometer (Promega, Madison, WI, USA), and used to construct libraries with the VAHTS^®^ Universal Plus DNA Library Prep Kit for MGI V2 (Vazyme Biotechnology, Nanjing, China). Paired-end sequencing was performed on the DNBSEQ-G99 platform (BGI Genomics, Shenzhen, China). The raw sequence data have been deposited in the Genome Sequence Archive in National Genomics Data Center, China National Center for Bioinformation/Beijing Institute of Genomics, Chinese Academy of Sciences (GSA: CRA047840).

Raw reads were quality-filtered with fastp v0.20.0 and merged using FLASH v1.2.7 [14,15]. High-quality sequences were clustered into operational taxonomic units (OTUs) at 97% similarity using UPARSE v7.1, with chimeras removed during clustering [16]. Representative sequences were classified against the SILVA 16S rRNA database (release 138) using the RDP Classifier v2.2 [17]. Alpha diversity was evaluated using richness, Chao1, and Shannon indices. Beta diversity was visualized by principal coordinate analysis based on Bray-Curtis distances, and group differences were assessed using PERMANOVA and ANOSIM. LEfSe was used to identify discriminatory taxa through a Kruskal–Wallis test (*p* < 0.05), pairwise Wilcoxon tests (*p* < 0.05), and linear discriminant analysis. Taxa with an LDA score > 3.0 were considered biomarkers.

### 2.7. Transcriptome Analysis

Total RNA was extracted from intestinal tissues using TRIzol Reagent (Invitrogen, Carlsbad, CA, USA). RNA integrity was assessed using an Agilent 2100 Bioanalyzer (Agilent Technologies, Santa Clara, CA, USA), and samples with an RNA integrity number ≥ 6.5 were used for library construction. Poly(A) mRNA was enriched using oligo(dT) magnetic beads, and strand-specific cDNA libraries were prepared following the manufacturer’s protocol. Libraries were sequenced on the Illumina HiSeq X Ten platform (Illumina, San Diego, CA, USA) to generate 150 bp paired-end reads. The raw sequence data have been deposited in the Genome Sequence Archive in National Genomics Data Center, China National Center for Bioinformation/Beijing Institute of Genomics, Chinese Academy of Sciences (GSA: CRA047837).

Raw reads were filtered using fastp v0.23.4 to remove adapters and low-quality sequences [14]. Clean reads were aligned to the grass carp reference genome (GCF_019924925.1) using STAR v2.7.5c [18]. Differential expression analysis was performed using DESeq2 v1.44.0 [19]. Genes with an absolute log_2_ fold change (|log_2_FC|) > 1 and a false discovery rate (FDR) < 0.05 were considered differentially expressed. Functional enrichment analyses of differentially expressed genes were performed using the Gene Ontology (GO) and Kyoto Encyclopedia of Genes and Genomes (KEGG) databases with GOATools v1.6.0 and KOBAS v 3.0, respectively [20]. GO terms and KEGG pathways with an FDR < 0.05 were considered significantly enriched. Gene set enrichment analysis (GSEA v1.26.0) was conducted using genes ranked by log_2_ fold change against KEGG and GO biological process, cellular component, and molecular function gene sets [21]. Gene sets with |NES| > 1, *p* < 0.05, and FDR q < 0.25 were considered significantly enriched [21].

### 2.8. Intestinal Histopathology

Intestinal segments fixed in 10% neutral-buffered formalin were dehydrated through a graded ethanol series, cleared in xylene, embedded in paraffin, and sectioned at 5 μm thickness. The sections were stained with hematoxylin and eosin (H&E) and examined under a light microscope for histopathological evaluation. All slides were coded and evaluated by a qualified pathologist under blinded conditions. Histopathological findings were assessed across all six sampled fish per treatment group.

### 2.9. Biochemical Assays

Frozen intestinal tissues (approximately 140 mg) were thawed on ice, weighed, and homogenized in ice-cold physiological saline at a 1:9 (*w*/*v*) ratio. The homogenates were centrifuged at 3000× *g* for 10 min at 4 °C, and the supernatants were collected for biochemical analyses. Total protein was quantified with a BCA Protein Assay Kit, and T-SOD, CAT activities, GSH, and MDA contents were measured using commercial kits according to the manufacturers’ instructions.

### 2.10. Statistical Analysis

All statistical analyses were performed using R software (version 4.3.3). The 48 and 96 h median lethal concentrations (LC_50_) and 95% confidence intervals (CI) were calculated using Probit regression analysis. Because exposure concentrations were nominal and were not analytically verified, the LC_50_ estimates are based on nominal concentrations. Biochemical data are presented as mean ± standard error (SE). Differences among treatment groups were evaluated by one-way analysis of variance (ANOVA), followed by Tukey’s HSD test. A *p* value < 0.05 was considered statistically significant.

## 3. Results

### 3.1. Acute Toxicity of Hexaflumuron

Mortality increased with both exposure concentration and time (Table 1 and Figure S1). No deaths occurred in the blank or solvent controls. Mortality remained low at concentrations of 4 and 6 mg/L during the first 48 h and increased thereafter. Exposure to 10 and 12 mg/L caused rapid mortality, and all fish died within 24 h at concentrations of 12 mg/L. Fish exposed to 8 mg/L displayed intermediate toxicity, with cumulative mortality increasing progressively over time. Probit analysis yielded 48 and 96 h LC_50_ values of 8.80 mg/L (95% CI: 7.87–9.82 mg/L) and 7.16 mg/L (95% CI: 5.81–8.46 mg/L), respectively.

Behavioral changes were observed before substantial mortality. During the first 12–24 h, fish exposed to concentrations below 8 mg/L exhibited reduced swimming activity, loss of equilibrium, and frequent ventral-up swimming despite relatively low mortality. These behavioral responses became more pronounced with increasing exposure concentration and time.

### 3.2. Intestinal Histopathological Alterations

**Figure 1:**
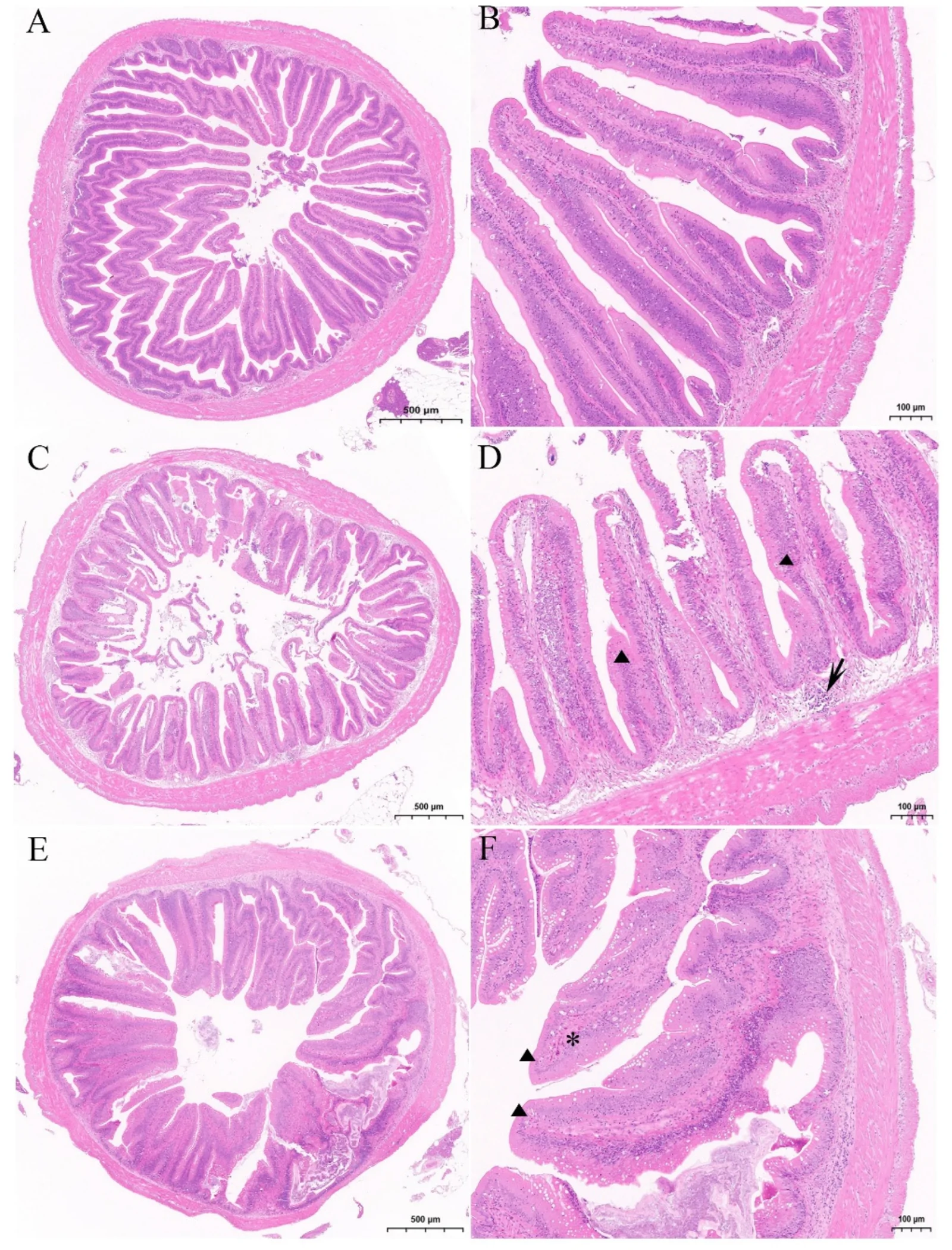
Hematoxylin and eosin-stained intestinal sections of grass carp following 96 h exposure to the solvent control, 0.07 mg/L hexaflumuron, or 0.72 mg/L hexaflumuron. (**A**,**B**) represent the solvent control group; (**C**,**D**) represent the low-concentration group (0.07 mg/L); and (**E**,**F**) represent the high-concentration group (0.72 mg/L). Triangles indicate epithelial necrosis, arrows indicate lymphocyte infiltration, and asterisks indicate capillary congestion.

Histopathological examination using H&E staining revealed focal intestinal alterations following hexaflumuron exposure (Figure 1). In the control group, the intestinal structure was well preserved, with abundant finger-shaped villi arranged regularly and normal muscle fiber morphology. Focal epithelial sloughing was observed sporadically in both control and exposed fish and was therefore not considered a treatment-specific lesion. In the low-concentration group, the villi remained abundant but were slightly disorganized, with focal epithelial necrosis (three out of six fish), widened spaces between the epithelium and lamina propria, and mild lymphocyte infiltration (one out of six fish). The high-concentration group showed irregular villus arrangement, focal epithelial necrosis (five out of six fish), sporadic capillary congestion (two out of six fish), and mild lymphocyte infiltration (three out of six fish).

### 3.3. Oxidative Stress and Antioxidant Responses

**Figure 2:**
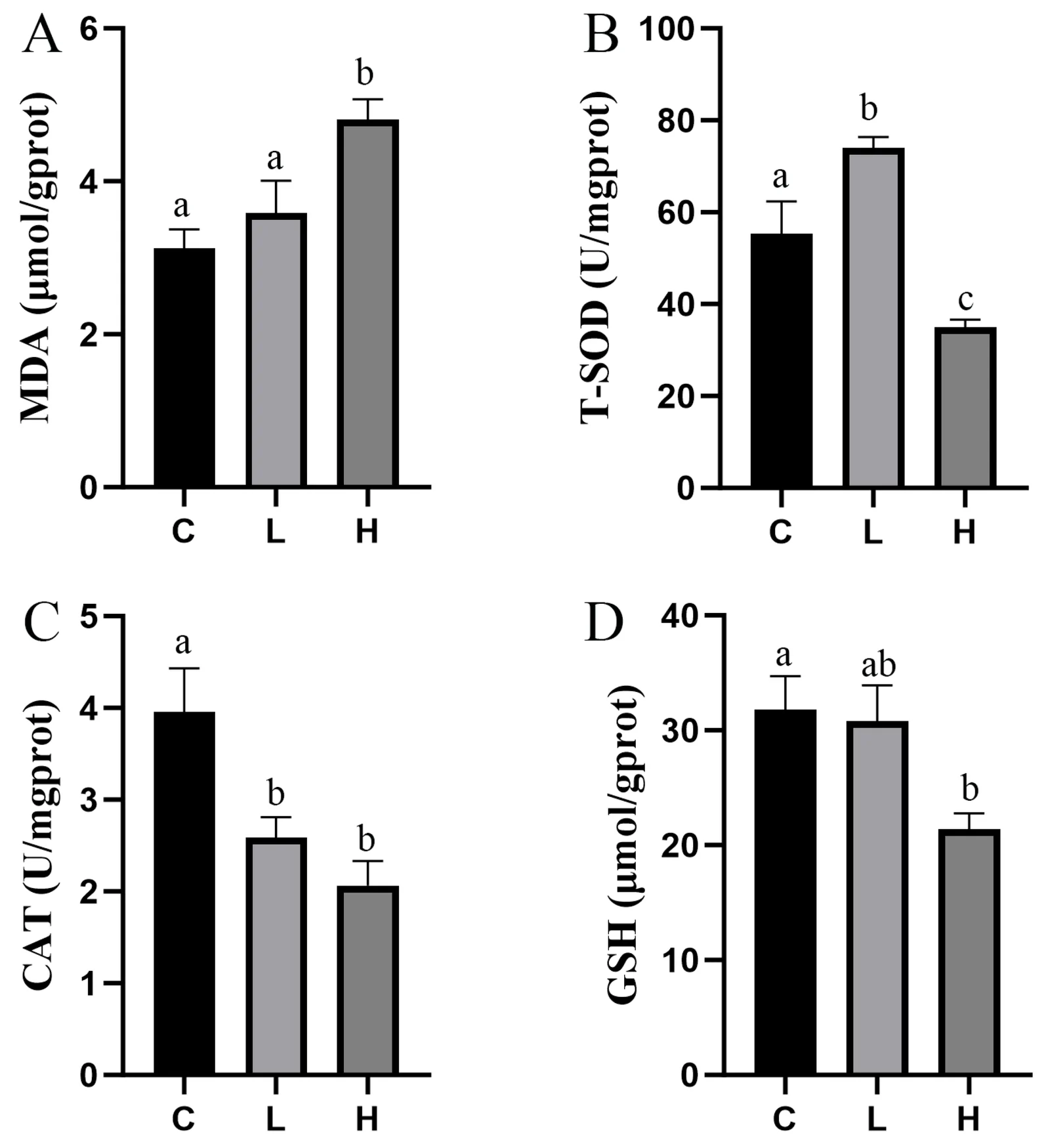
Changes in intestinal antioxidant status and lipid peroxidation in grass carp following 96-h hexaflumuron exposure. (**A**) Malondialdehyde (MDA) content; (**B**) total superoxide dismutase (T-SOD) activity; (**C**) catalase (CAT) activity; and (**D**) reduced glutathione (GSH) content. Values are expressed as mean ± standard error (SE) (N = 3 tanks ∗ 2 per tank). Bars sharing different letters indicate statistically significant differences among all treatments (one-way ANOVA followed by Tukey’s HSD post hoc test, *p* < 0.05). C, solvent control; L, 0.07 mg/L; H, 0.72 mg/L.

Hexaflumuron exposure induced obvious oxidative stress in the intestine of grass carp (Figure 2). The content of MDA, a marker of lipid peroxidation, increased across exposure concentrations and was significantly higher than the control in the high-concentration group (*p* = 0.008). In contrast, antioxidant defense parameters exhibited distinct responses across the exposure groups. T-SOD activity was significantly elevated in the low-concentration group (*p* = 0.028) but declined below the control level in the high-concentration group (*p* = 0.016). CAT activity and GSH levels decreased progressively with increasing nominal exposure concentration, with both parameters showing significant reductions in the high-concentration group (*p* = 0.005 and *p* = 0.038, respectively). These changes were consistent with altered antioxidant regulation and increased lipid peroxidation under the exposure conditions.

### 3.4. Gut Microbiota Responses to Hexaflumuron Exposure

#### 3.4.1. Diversity Analysis

**Figure 3:**
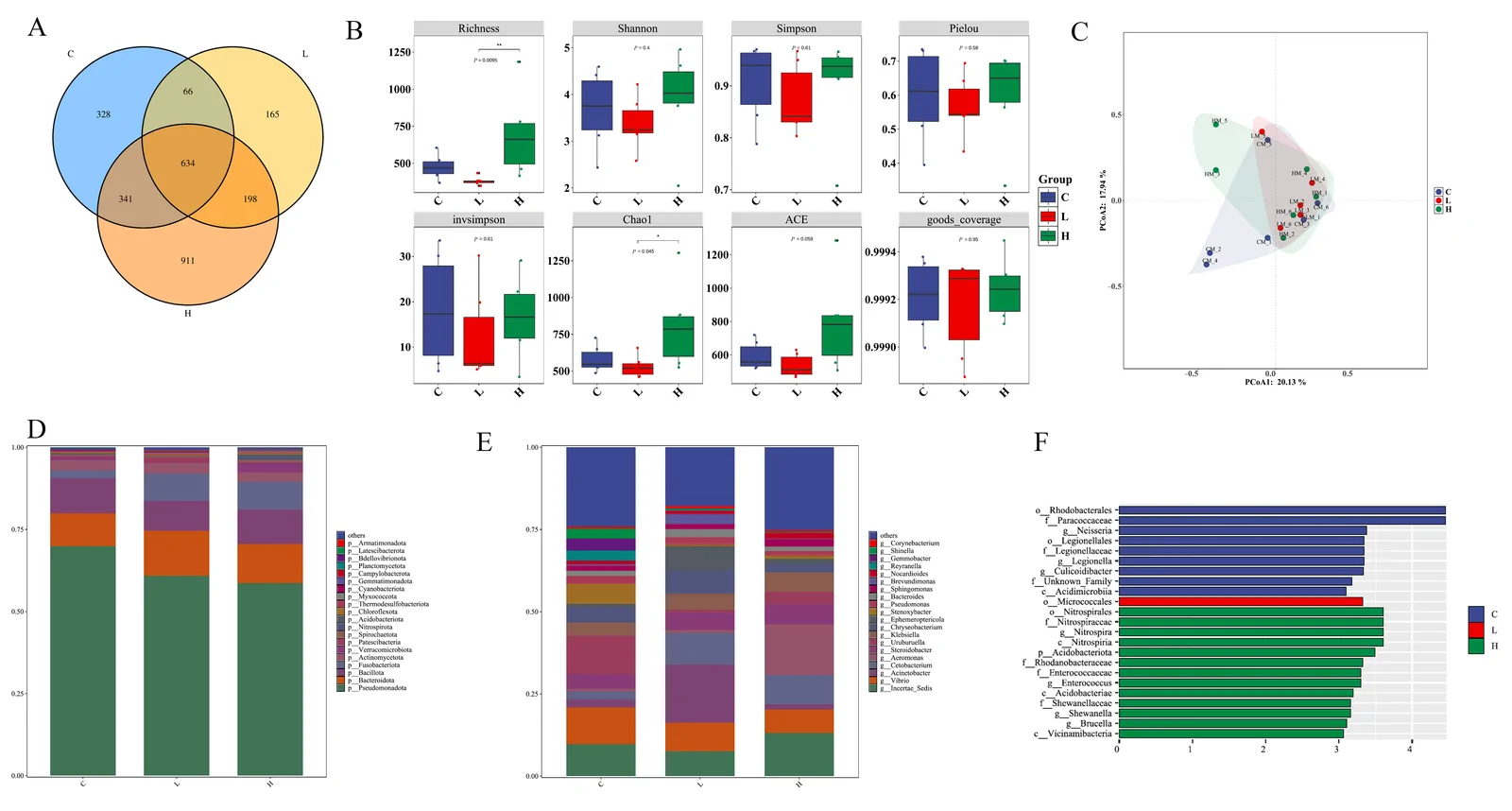
Effects of 96 h hexaflumuron exposure on the intestinal microbiota of grass carp (n = 6 fish per group (2 fish per tank)). (**A**) Shared and unique operational taxonomic units among groups; (**B**) alpha-diversity indices; (**C**) principal coordinate analysis based on Bray-Curtis distances; (**D**) phylum-level relative abundance; (**E**) genus-level relative abundance; and (**F**) LEfSe biomarkers (LDA score > 3.0, *p* < 0.05). C, solvent control; L, 0.07 mg/L; H, 0.72 mg/L.

High-throughput sequencing of the V3-V4 region of the bacterial 16S rRNA gene was performed to characterize the intestinal microbiota responses to hexaflumuron exposure. A total of 18 intestinal content samples from the control (C), low-concentration (L), and high-concentration (H) groups were analyzed. After quality filtering, 2,893,597 high-quality sequences were obtained. Good’s coverage exceeded 99.9% for all samples, indicating sufficient sequencing depth to represent the majority of bacterial communities. A total of 9805 operational taxonomic units were identified, of which 634 OTUs were shared among all groups (Figure 3A).

Alpha diversity showed a non-monotonic response to hexaflumuron (Figure 3B). Compared with the control group, the low-concentration group exhibited decreased microbial richness and Shannon diversity. In contrast, microbial richness increased in the high-concentration group, accompanied by elevated Chao1 values and increased variability among biological replicates. Principal coordinate analysis based on Bray–Curtis distances showed partial group separation (Figure 3C), but neither PERMANOVA (R^2^ = 0.145, *p* = 0.167) nor ANOSIM (*p* > 0.05) detected a significant difference in overall community structure. These results suggested that short-term hexaflumuron exposure had limited impacts on the global structure of the intestinal microbiota.

#### 3.4.2. Microbial Community Shifts

Although the overall community structure remained relatively stable, substantial alterations in specific bacterial taxa were observed at both phylum and genus levels. At the phylum level, Pseudomonadota, the dominant bacterial group in the control fish, showed a gradual decline with increasing hexaflumuron concentration, decreasing from 69.88% in the control group to 60.91% and 58.61% in the low- and high-concentration groups, respectively. In contrast, the relative abundances of Fusobacteriota and Verrucomicrobiota increased markedly in the high-concentration group, increasing from 2.35% to 8.50% and from 0.77% to 2.85%, respectively.

At the genus level, distinct bacterial shifts were observed across the hexaflumuron exposure groups. The relative abundance of *Acinetobacter* was markedly elevated in the low-concentration group from 2.44% (control) to 17.75% but declined to 1.61% at the high exposure concentration. In contrast, the genus *Aeromonas*, which includes several well-known opportunistic pathogens of fish, remained relatively stable in the control and low-concentration groups (0.74% and 0.93%, respectively), but increased markedly to 15.40% in the high-concentration group (Figure 3D,E).

#### 3.4.3. Microbial Biomarkers

LEfSe analysis identified bacterial biomarkers associated with different exposure conditions (Figure 3F). The control group was enriched with several commensal bacterial taxa, including members of Rhodobacterales and Neisseria, whereas the high-concentration group was characterized by significant enrichment of Nitrospirota (LDA = 3.61), Acidobacteriota, and members of the Enterococcaceae family.

### 3.5. Transcriptomic Responses of the Intestine to Hexaflumuron Exposure

#### 3.5.1. Transcriptome Sequencing and DEGs

RNA sequencing was performed to characterize the intestinal transcriptional responses of grass carp following hexaflumuron exposure. A total of 18 intestinal samples were subjected to transcriptome sequencing using the Illumina platform. All samples exhibited high sequencing quality, with Q30 values exceeding 97.67%. The clean reads showed high mapping efficiency against the grass carp reference genome, with alignment rates ranging from 94.83% to 98.16%, confirming the reliability of the sequencing data for subsequent transcriptomic analyses (Table S1).

Hexaflumuron exposure was associated with a concentration-related expansion of the intestinal transcriptional response. Compared with the control group, the low-concentration group (L vs. C) exhibited 1226 DEGs, including 665 upregulated and 561 downregulated genes, whereas the high-concentration group (H vs. C) exhibited 5496 DEGs, including 3125 upregulated and 2371 downregulated genes. Direct comparison of the two exposure groups (H vs. L) identified 4846 DEGs, comprising 2339 upregulated and 2507 downregulated genes. Notably, the most strongly downregulated transcripts in H vs. C were the mitochondrially encoded genes ATP8, ATP6, COX1, COX2, and COX3 (log_2_ fold changes of approximately −4.70 to −5.17). ATP6, ATP8, CYTB, and COX3 were also significantly downregulated in L vs. C, but with smaller changes (approximately −1.10 to −1.71), indicating that suppression of mitochondrial transcripts intensified at the higher concentration (Table S2).

#### 3.5.2. GO Enrichment Analysis

**Figure 4:**
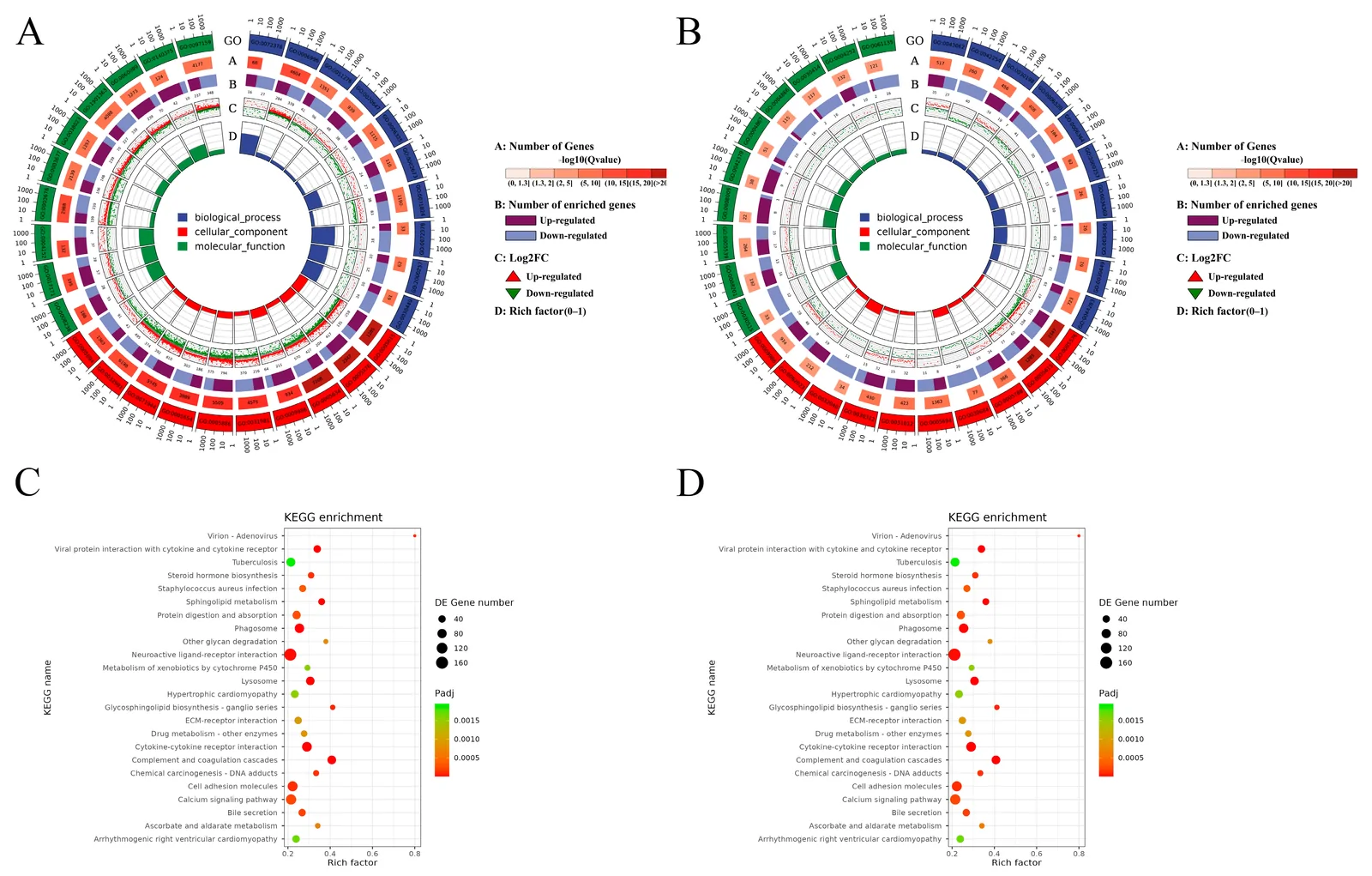
Intestinal transcriptomic responses of grass carp following 96 h hexaflumuron exposure (n = 6 fish per group (2 fish per tank)). (**A**,**B**) GO enrichment of differentially expressed genes in H vs. C and L vs. C; (**C**,**D**) KEGG enrichment of differentially expressed genes in H vs. C and L vs. C. C, solvent control; L, 0.07 mg/L; H, 0.72 mg/L.

GO enrichment analysis revealed divergent functional responses in H vs. C (Figure 4A). Upregulated DEGs were enriched in cell activation, immune response, leukocyte activation, leukocyte-mediated immunity, as well as RNA- and nucleic-acid-related processes. Enriched cellular-component terms included the plasma membrane, cell periphery, extracellular region, and nuclear lumen, while enriched molecular functions included signaling-receptor activity, molecular-transducer activity, nucleic-acid binding, and serine-hydrolase activity. In contrast, downregulated genes were associated with protein-activation cascades, fibrin-clot formation, regulation of complement activation, and coagulation-related processes. For cellular-component terms, the endomembrane system, endosomes, and intracellular vesicles were enriched. For molecular-function terms, serine-type endopeptidase-inhibitor and cytokine-binding activities were enriched (Table S3).

In L vs. C, upregulated DEGs were enriched in extracellular-matrix and extracellular-structure organization, vascular development, regulation of cell migration, and chemotaxis (Figure 4B). Chemokine activity and chemokine-receptor binding were the leading molecular-function terms. Downregulated DEGs were enriched in ribosome biogenesis, rRNA processing, ribonucleoprotein-complex biogenesis, amino-acid metabolism, and small-molecule metabolism. Preribosomal and nucleolar components and snoRNA-binding, peptidase-inhibitor, and aminoacyl-tRNA-ligase activities were also suppressed. The low-concentration response was therefore characterized by extracellular remodeling and chemotactic signaling together with suppression of protein synthesis and metabolic processing (Table S3).

#### 3.5.3. KEGG Pathway Analysis

KEGG analysis of upregulated DEGs in H vs. C showed prominent enrichment of lysosome (59 genes), cytokine-cytokine receptor interaction (76 genes), phagosome (69 genes), cell adhesion molecules (82 genes), antigen processing and presentation (38 genes), and natural killer cell-mediated cytotoxicity (42 genes) (Figure 4C). Sphingolipid metabolism was also significantly enriched. Together, these pathways indicated increased immune signaling, phagolysosomal processing, cell–cell interaction, and membrane–lipid remodeling (Table S4).

Conversely, downregulated DEGs in H vs. C were most strongly enriched in complement and coagulation cascades (44 genes), followed by the spliceosome (28 genes), oxidative phosphorylation (24 genes), aminoacyl-tRNA biosynthesis (11 genes), and ribosome biogenesis in eukaryotes (16 genes) (Figure 4C). Several amino acid metabolic pathways were also suppressed. These results indicated simultaneous activation of cellular immune and degradative programs and inhibition of complement-associated defense, RNA processing, translation, and mitochondrial energy metabolism.

In L vs. C, upregulated DEGs were enriched in protein digestion and absorption (16 genes), cytokine–cytokine receptor interaction (18 genes), ECM–receptor interaction (11 genes), and complement and coagulation cascades (11 genes) (Figure 4D). The complement and coagulation pathway also contained 24 downregulated genes, indicating bidirectional gene-level remodeling. Downregulated DEGs were further enriched in ribosome biogenesis in eukaryotes (18 genes), aminoacyl-tRNA biosynthesis (10 genes), oxidative phosphorylation (16 genes), and multiple amino-acid metabolic pathways (Table S4). Translational, proteostatic, and mitochondrial metabolic suppression therefore accompanied extracellular-matrix remodeling and immune signaling in the low-concentration group.

#### 3.5.4. GSEA Analysis

**Figure 5:**
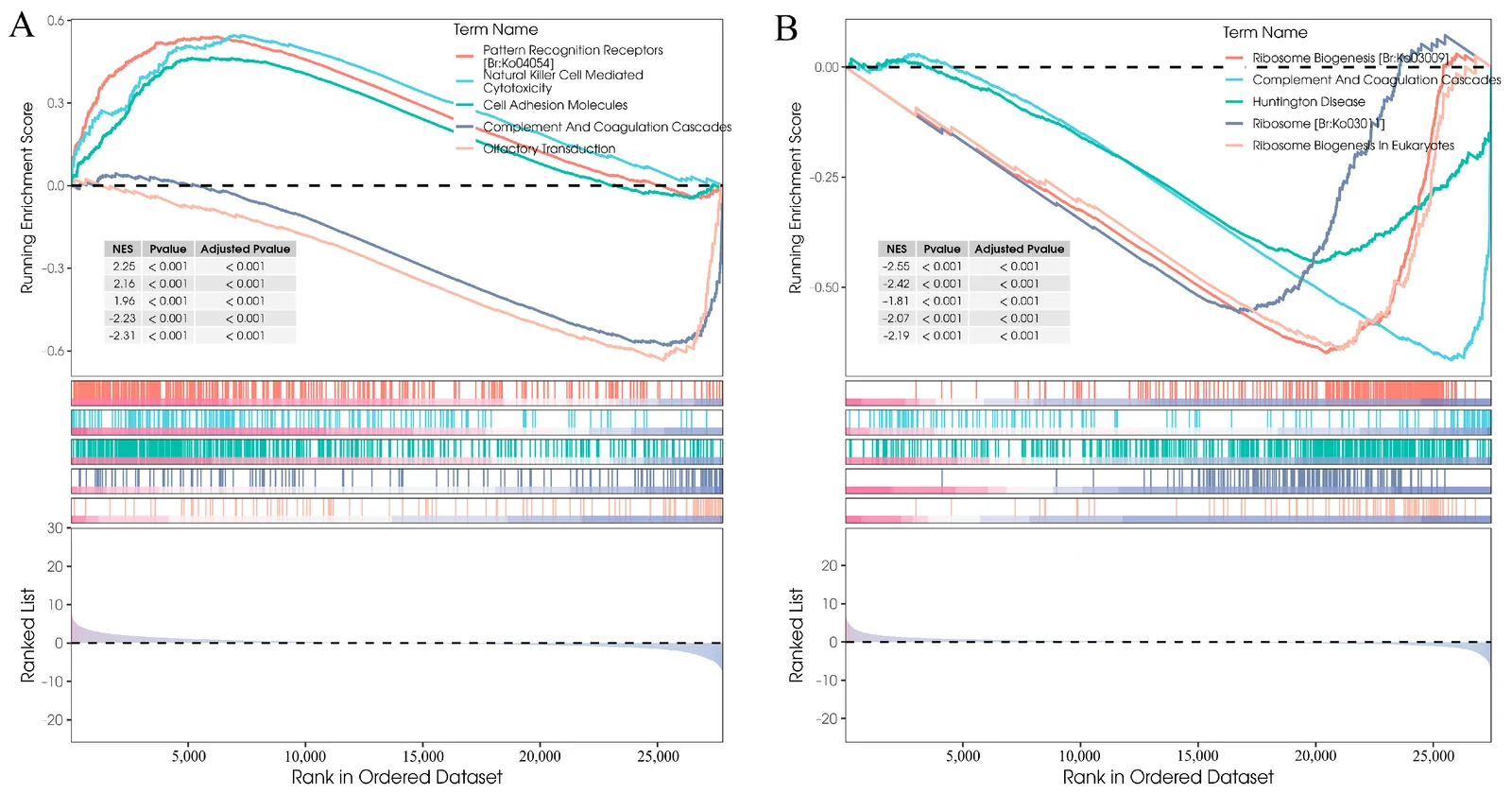
Gene set enrichment analysis of intestinal transcriptomic responses following 96-h hexaflumuron exposure (n = 6 fish per group (2 fish per tank)). Representative KEGG enrichment profiles are shown for H vs. C (**A**) and L vs. C (**B**). C, solvent control; L, 0.07 mg/L; H, 0.72 mg/L.

GSEA revealed coordinated pathway-level responses in H vs. C. Positively enriched KEGG gene sets included pattern-recognition receptors (NES = 2.251), viral protein interaction with cytokines and cytokine receptors (NES = 2.237), antigen processing and presentation (NES = 2.163), natural killer cell-mediated cytotoxicity (NES = 2.155), and the intestinal immune network for IgA production (NES = 2.005) (Figure 5A). GO GSEA identified positive enrichment of neutrophil activation and degranulation, leukocyte adhesion and extravasation, chemokine-mediated signaling, and lymphocyte migration. Complement and coagulation cascades were negatively enriched (NES = −2.228), together with ribosomal and amino acid metabolic pathways. Ribosome biogenesis (NES = −2.213), rRNA processing (NES = −2.203), regulation of complement activation (NES = −2.165), mitochondrial translation (NES = −2.104), and mitochondrial protein-containing complexes (NES = −2.103) were also negatively enriched (Table S5).

In L vs. C, natural killer cell-mediated cytotoxicity, antigen processing and presentation, the intestinal immune network for IgA production, and several immune-cell migration gene sets showed positive enrichment at the predefined GSEA threshold. The strongest negative enrichment signatures were ribosome biogenesis (NES = −2.552), complement and coagulation cascades (NES = −2.415), proteasome pathways (NES = −2.095 to −2.125), oxidative phosphorylation (NES = −2.005), rRNA processing (NES = −2.513), mitochondrial protein-containing complexes (NES = −2.436), and regulation of complement activation (NES = −2.301) (Figure 5B). Across both exposure concentrations, ribosome production, protein homeostasis, mitochondrial bioenergetic programs, and complement-associated functions were suppressed, while high-concentration exposure involved a broader set of inflammatory and immune-effector pathways (Table S5).

## 4. Discussion

### 4.1. Acute Toxicity and Behavioral Responses

Benzoylurea insecticides have received growing attention as candidate antiparasitic agents in aquaculture [3,5]. However, their potential adverse effects on non-target aquatic organisms remain poorly understood. In this study, hexaflumuron exhibited clear time- and concentration-associated mortality in grass carp, with 48 and 96 h LC_50_ values of 8.80 and 7.16 mg/L, respectively. The lower LC_50_ with prolonged exposure was consistent with greater toxicity at longer exposure duration. The 96 h LC_50_ was closely comparable to the value of 7.19 mg/L previously reported for Nile tilapia, suggesting broadly similar acute sensitivity between the two freshwater fish species [9]. Nevertheless, differences in fish size, developmental stage, water quality, and chemical formulation should be considered when comparing toxicity thresholds among studies.

Reduced swimming, loss of equilibrium, and ventral-up swimming occurred before extensive mortality and became more frequent with increasing concentration and duration. These responses indicated early disturbance of physiological or neuromuscular function and may serve as sensitive warning endpoints during acute exposure. Similar behavioral alterations have been reported in hexaflumuron-exposed Nile tilapia together with oxidative and stress-related changes [9]. Such behavioral impairment might reduce feeding, predator avoidance, and environmental adaptability even at low mortality levels.

Although benzoylurea insecticides are designed primarily to disrupt chitin synthesis and molting in arthropods, their arthropod-selective mode of action does not ensure complete safety in fish [22,23]. Hexaflumuron is highly active against salmon-louse larvae, with substantially greater molting-inhibitory potency than several other chitin synthesis inhibitors [2]. Therefore, its antiparasitic efficacy should be evaluated together with host toxicity when determining treatment concentrations and exposure duration in aquaculture.

### 4.2. Intestinal Oxidative Stress and Tissue Alterations

Short-term sublethal exposure was associated with focal intestinal lesions, including villus disorganization, focal epithelial necrosis, separation between the epithelium and lamina propria, lymphocyte infiltration, and occasional capillary congestion. Focal epithelial sloughing was also observed in control and exposed fish and was therefore considered a nonspecific background and not a treatment-specific lesion. Despite these localized alterations, the overall intestinal architecture was largely preserved, with abundant finger-shaped villi remaining structurally intact. The histological findings were focal and did not involve extensive architectural destruction during the 96 h exposure [24].

The histological alterations were accompanied by altered intestinal redox status. MDA levels increased with exposure concentration, whereas CAT activity and GSH content progressively declined [25]. T-SOD activity increased at the low concentration but decreased below the control level at the high concentration. The elevation of T-SOD under low exposure might reflect an adaptive response to increased superoxide production [26]. However, the concurrent decline in CAT and GSH might limit the removal of hydrogen peroxide and other oxidants. At the higher concentration, reductions in T-SOD, CAT, and GSH suggested that antioxidant defenses were insufficient to counteract the oxidative challenge, thereby promoting lipid peroxidation [27]. A similar pattern has been reported in Nile tilapia, in which hexaflumuron exposure was associated with reduced antioxidant capacity and increased MDA levels [9]. Prolonged exposure to 0.72 mg/L also induced antioxidant-immune disturbances and tissue damage, suggesting that the early redox alterations observed in grass carp might become more pronounced under repeated or extended exposure.

The relatively pronounced biochemical response compared with the histological changes suggested that antioxidant and lipid-peroxidation indicators may detect intestinal stress before extensive structural damage becomes apparent. Oxidative imbalance may therefore be an early component of the intestinal response to hexaflumuron, and may contribute to subsequent impairment of epithelial integrity, cellular metabolism, and tissue repair.

### 4.3. Shifts in Gut Microbial Communities

Hexaflumuron exposure induced a nonlinear response in intestinal microbial diversity. Richness and Shannon diversity decreased in the low-concentration group, whereas richness and Chao1 values increased in the high-concentration group. Increased microbial richness under high exposure should not necessarily be interpreted as a beneficial ecological shift, because chemical stress might alter community structure and allow previously low-abundance taxa to expand [28]. PCoA based on Bray–Curtis distances showed partial separation among the control and exposure groups. However, neither PERMANOVA nor ANOSIM detected significant differences in overall community composition. These results indicated that 96 h hexaflumuron exposure did not cause a statistically significant restructuring of the entire intestinal microbiota. Instead, the microbial response was mainly reflected by selective alterations of specific bacterial populations. The absence of significant β-diversity differences might also reflect the considerable interindividual variation observed, particularly in the high-concentration group.

At the phylum level, Pseudomonadota remained dominant but declined progressively with increasing exposure concentration, whereas Fusobacteriota and Verrucomicrobiota increased in the high-concentration group. The reduction in Pseudomonadota suggested disruption of the core dominant microbial community and redistribution of intestinal ecological niches. Increased Fusobacteriota might reflect altered fermentative metabolism and nutrient utilization, while enrichment of Verrucomicrobiota might indicate changes in mucin availability and the mucosal environment [29]. The concurrent decline in Pseudomonadota and expansion of *Aeromonas* suggested substantial compositional replacement within this phylum, which might weaken microbial homeostasis and colonization resistance [30,31].

More distinct changes occurred at the genus level. *Acinetobacter* increased markedly in the low-concentration group but declined in the high-concentration group. This pattern might reflect differential sensitivity to exposure intensity, although the underlying ecological mechanism remains uncertain. *Aeromonas* increased from below 1% in the control and low-concentration groups to 15.40% in the high-concentration group. Several *Aeromonas* species are recognized as opportunistic pathogens of cultured fish [32,33]. Their enrichment is consistent with a shift in the intestinal environment under high-concentration exposure that may favor potentially harmful bacteria [34,35]. The simultaneous occurrence of epithelial alterations, oxidative stress, lymphocyte infiltration, and immune-related transcriptional responses provided a coherent biological context for this microbial shift. Nevertheless, genus-level sequencing cannot distinguish pathogenic from nonpathogenic *Aeromonas* strains, and species identification and virulence-gene analysis would be required to confirm increased disease risk. LEfSe analysis further identified Rhodobacterales and Neisseria as biomarkers of the control group, whereas Nitrospirota, Acidobacteriota, and Enterococcaceae were enriched under high-concentration exposure. These findings further supported selective changes in bacterial lineages despite the absence of significant differences in overall β-diversity. The microbiome data therefore indicated taxon-specific microbial shifts associated with intestinal stress, although they did not establish whether the microbial changes were a cause or consequence of host responses.

### 4.4. Transcriptomic Responses to Hexaflumuron

The transcriptomic analysis indicated that the low-concentration response was characterized by coordinated suppression of ribosome biogenesis, rRNA processing, aminoacyl-tRNA biosynthesis, proteasome pathways, oxidative phosphorylation, and amino-acid metabolism. The downregulation of ATP6, ATP8, CYTB, and COX3 further supported an early reduction in mitochondrial transcriptional activity [8]. Collectively, these changes were consistent with reduced biosynthetic capacity and altered protein homeostasis, which might affect epithelial renewal, digestive-enzyme production, and tissue repair. In parallel, extracellular-matrix organization, ECM-receptor interaction, chemokine activity, and cell-migration processes were enriched, which indicated that structural remodeling and immune-cell recruitment were initiated even at the lower concentration [36]. Enrichment of the protein digestion and absorption pathway should therefore not be interpreted solely as enhanced digestive function, because many genes assigned to this pathway encode collagen and extracellular-matrix components.

At the high concentration, pattern-recognition, cytokine-receptor, lysosome, phagosome, antigen-presentation, natural killer cell cytotoxicity, neutrophil activation, and chemokine-mediated migration programs were positively enriched. These enrichment signatures supported recruitment and activation of intestinal immune cells and are consistent with the lymphocyte infiltration observed histologically [37]. Complement and coagulation cascades and regulation of complement activation were negatively enriched, together with spliceosomal, ribosomal, aminoacyl-tRNA, mitochondrial-translation, and oxidative-phosphorylation pathways. This expression profile described an immune-dysregulated state marked by enhanced inflammatory and phagocytic signaling, impaired complement-mediated defense, and widespread suppression of cellular biosynthesis and energy metabolism.

Mitochondrial perturbation was one of the most consistent molecular features across exposure concentrations. The concentration-associated downregulation of mitochondrially encoded ATP synthase and cytochrome c oxidase subunits, together with negative enrichment of oxidative phosphorylation, mitochondrial translation, and mitochondrial protein-containing complexes, is consistent with reduced electron transport and ATP-generating capacity [38,39]. These findings were consistent with the increased lipid peroxidation and impaired antioxidant defenses observed biochemically, although direct mitochondrial functional measurements were not performed. Nevertheless, ATP content, respiratory-complex activity, mitochondrial membrane potential, and mitochondrial ROS were not measured directly; therefore, the data supported transcriptional suppression of mitochondrial bioenergetic programs but did not establish mitochondrial functional failure.

### 4.5. Integrated Interpretation and Limitations

Integration of the different indicators indicated coordinated intestinal responses across the nominal exposure groups. At the low concentration, suppression of protein synthesis, protein turnover, amino-acid metabolism, mitochondrial bioenergetics, and complement-associated functions occurred alongside extracellular matrix remodeling and modest chemotactic signaling. At the high concentration, mitochondrial transcripts were more strongly downregulated, antioxidant capacity declined, lipid peroxidation increased, and transcriptional responses expanded to include pattern-recognition, cytokine, phagolysosomal, antigen-presentation, and leukocyte-migration programs. These changes coincided with focal mucosal lesions and selective enrichment of *Aeromonas* and other exposure-associated bacterial taxa. A plausible interpretation is that metabolic constraint and redox imbalance might reduce the capacity of the intestinal mucosa to maintain and repair itself, while inflammatory recruitment and complement-related changes might alter host defense and favor taxon-specific microbial expansion. However, because internal hexaflumuron concentrations were not measured, these relationships should be interpreted as exposure-associated, not as evidence of a quantified tissue-dose response. The results also indicated that acute mortality does not capture all measurable biological responses to hexaflumuron exposure. Biochemical, transcriptional, histological, and microbial responses were detectable at nominal concentrations corresponding to 1/100 and 1/10 of the 96 h LC_50_. Safety assessment should therefore consider sublethal effects, repeated treatment, exposure duration, post-treatment recovery, and the relationship between antiparasitic efficacy and host physiological responses.

Several limitations should be considered in this study. Exposure concentrations were nominal and were not verified in water or fish tissues, so the study did not establish an internal tissue-dose–response relationship. Focal epithelial sloughing was observed in control as well as exposed fish and was therefore not considered a treatment-specific lesion; interpretation instead focused on focal epithelial necrosis and other exposure-associated structural or inflammatory changes. The 96 h acute design followed a predefined mortality endpoint but did not include a recovery phase, so the reversibility of severe behavioral or physiological effects was not assessed. In addition, although this study focused on intestinal responses, waterborne exposure might also affect other major organs, including the gills, liver, and kidney. The present data therefore did not establish the intestine as a primary or exclusive target organ and should not be interpreted as a complete assessment of systemic toxicity. RNA sequencing cannot distinguish transcriptional changes within epithelial cells from changes caused by altered immune-cell abundance [40]. Mitochondrial impairment, translational suppression, complement dysfunction, and barrier injury were inferred from expression patterns, while 16S sequencing lacked species-level and functional resolution. Future studies should include verified exposure concentrations, tissue residue measurements, recovery experiments, comparative multi-organ assessment, ATP and mitochondrial-respiration measurements, functional assessments of intestinal barrier integrity, validation of representative mitochondrial, complement, and cytokine genes, and metagenomic or metabolomic profiling.

## 5. Conclusions

Hexaflumuron exposure was associated with concentration- and time-dependent acute mortality and early behavioral responses in grass carp. Sublethal 96 h exposure was associated with focal intestinal lesions, altered redox homeostasis, and changes in selected bacterial taxa without significantly changing the overall gut microbial community structure. Transcriptomic analysis further revealed coordinated changes in cellular biosynthesis, mitochondrial-related pathways, and immune regulation, with broader effects at the higher exposure concentration. Collectively, the results indicated that sublethal hexaflumuron exposure disrupted intestinal homeostasis through interconnected metabolic, redox, immune, tissue-remodeling, and microbial responses, which provided a mechanistic basis for ecological risk assessment and safer application in aquaculture.

## Figures and Tables

**Table 1:** Acute toxicity of hexaflumuron in grass carp (*Ctenopharyngodon idella*). Values are mean ± SE (n = 3 tanks).

Concentration (mg/L)	No. of Fish/Tank	No. Dead Fish (Mean ± SE)			
		24 h	48 h	72 h	96 h
Blank control	10	0.00	0.00	0.00	0.00
Solvent control	10	0.00	0.00	0.00	0.00
4	10	0.00	0.00	1.00 ± 0.58	2.33 ± 0.33
6	10	0.33 ± 0.33	1.33 ± 0.33	2.00 ± 1.00	3.33 ± 0.33
8	10	1.33 ± 0.67	1.67 ± 0.33	4.00 ± 0.58	4.67 ± 0.67
10	10	5.33 ± 0.67	7.67 ± 1.20	8.33 ± 0.88	8.33 ± 0.88
12	10	10.00	10.00	10.00	10.00

## Data Availability

The datasets used and analyzed during the current study are available from the corresponding author upon reasonable request. The raw 16S rRNA gene sequencing and transcriptome data have been deposited in the Genome Sequence Archive (GSA) at the National Genomics Data Center, China National Center for Bioinformation/Beijing Institute of Genomics, Chinese Academy of Sciences, under accession numbers CRA047840 (16S rRNA gene sequencing) and CRA047837 (transcriptome), respectively.

## Supplementary Materials

The following supporting information can be downloaded at: https://www.mdpi.com/article/10.3390/ani16193056/s1, Figure S1: Mortality of grass carp (*Ctenopharyngodon idella*) exposed to different concentrations of hexaflumuron over 96 h. Table S1: Summary of transcriptome sequencing data and read alignment rates; Table S2: Differentially expressed genes identified in the different group comparisons; Table S3: Gene Ontology enrichment results for differentially expressed genes in the different group comparisons; Table S4: Kyoto Encyclopedia of Genes and Genomes pathway enrichment results for differentially expressed genes in the different group comparisons; Table S5: Gene set enrichment analysis results for the different group comparisons.
